# Comparing the Rates of Further Resection After Intraoperative MRI Visualisation of Residual Tumour Between Brain Tumour Subtypes: A 17-Year Single-Centre Experience

**DOI:** 10.3390/brainsci15010045

**Published:** 2025-01-05

**Authors:** Daniel Madani, R. Dineth Fonseka, Sihyong Jake Kim, Patrick Tang, Krishna Muralidharan, Nicholas Chang, Johnny Wong

**Affiliations:** Department of Neurosurgery, Royal Prince Alfred Hospital, Sydney 2050, Australiapatrick.tang@health.nsw.gov.au (P.T.); johnny.hy.wong@gmail.com (J.W.)

**Keywords:** intraoperative imaging, MRI, brain tumour, glioma

## Abstract

BACKGROUND: Maximal safe resection is the objective of most neuro-oncological operations. Intraoperative magnetic resonance imaging (iMRI) may guide the surgeon to improve the extent of safe resection. There is limited evidence comparing the impact of iMRI on the rates of further resection between tumour types. AIM: To investigate the impact of iMRI on the rate of further resection following visualisation of residual tumour. METHODS: A retrospective cohort study identified all intracranial tumour operations performed in the 1.5 T iMRI machine of a single centre (2007–2023). Patients were identified using SurgiNet and were grouped according to their histopathological diagnosis in accordance with the WHO 2021 classification. The primary outcome was the rate of reoperation due to iMRI visualisation of residual tumours. RESULTS: A total of 574 cases were identified, including 152 low-grade gliomas (LGG), 108 high-grade gliomas (HGG), 194 pituitary neuroendocrine tumours (PitNETs), 15 metastases, and 6 meningiomas. Further resection following iMRI visualisation occurred in 45% of LGG cases, 47% of HGG cases, 29% of PitNET cases, and no meningioma or metastasis cases. Chi-square analysis showed that the rate of further resection after iMRI use across 2018–2023 was significantly higher than that across 2007–2012 (46% versus 33%, *p* = 0.036). CONCLUSION: Intraoperative MRI for guiding further resection was most useful in cases of LGG and HGG, possibly reflecting the difficulty of differentiating these tumour types from normal brain tissue. In addition, there was increased reliance on iMRI over time, which may represent our surgeons becoming accustomed to its use.

## 1. Introduction

The integration of intraoperative magnetic resonance imaging (iMRI) into surgical workflow represents a significant development in neurosurgical navigation. Intraoperative MRI offers real-time imaging during surgery, enabling precise and dynamic visualisation of tumour margins and critically important neurological structures [1]. Thus, iMRI may play a role in ensuring maximal safe resection and minimising residual abnormal tissue, an important prognostic factor underpinning patient outcomes following brain tumour resection [2]. This appears particularly relevant for the resection of brain tumours such as high-grade gliomas (HGG) that often display ill-defined borders that are difficult to appreciate at the macroscopic level [3]. For such cases, intraoperative MRIs may guide surgical resection of tumours near eloquent brain structures while preserving critical neurological function and improving patient survival [4]. Furthermore, the integration of functional MRI mapping offers an additional intra-operative solution to the current gold standard of invasive cortical stimulation [4]. Additionally, intraoperative MRI scanners have advanced from low-field to high-field strength systems, 0.15 T and 3 T, respectively [5]. Higher field strength is associated with superior image quality due to a higher signal-to-noise ratio and higher image acquisition speed [6]. As such, the quality of imaging available to surgeons intra-operatively has become comparable to the quality of imaging obtained in a non-operative MRI setting [5].

The literature generally supports the view that increasing the extent of resection without causing unacceptable deficits improves survival outcomes. Sanai et al. (2011) [7] conducted a retrospective study involving 500 patients with glioblastoma (GBM) and demonstrated a significant survival benefit with even a subtotal resection of 78%. Survival improved progressively, with the greatest benefit observed in the 95–100% extent of resection category. In low-grade gliomas (LGG), Smith et al. [8] conducted a retrospective study involving 216 patients, demonstrating that those with at least a 90% extent of resection had significantly higher overall survival rates—97% at 5 years and 91% at 8 years—compared to 76% and 60%, respectively, in patients with less than 90% resection. There are similar benefits to improving the extent of resection in other types of tumours; Brochier et al. (2010) [9] performed a retrospective study (*n* = 142) and noted that 24% (10 out of 42) of patients who had complete macroscopic resection of pituitary adenomas had recurrence after a mean follow-up period of about 7 years, compared to 47% (47 out of 100) of patients with a surgical remnant. Hence, it is anticipated that iMRI will improve survival outcomes if the technology allows surgeons to improve the extent of resection.

In addition, iMRI serves as a solution to dynamic changes that occur to the natural and pathological anatomy of the brain over the course of an operation. This phenomenon has been described in the literature as “brain shift”, whereby the brain undergoes intra-operative deformation due to tumour resection, removal of parts of the cranial cavity (craniotomy), oedema, haemorrhage and cerebrospinal fluid (CSF) drainage [10,11]. Displacement due to a brain shift of more than 10mm is reported to occur within one hour after the dural opening [12]. Consequently, the accuracy of traditional stereotactic neuronavigation diminishes throughout an operation. Intraoperative MRIs enable surgeons to acquire real-time images, accounting for dynamic changes and thus maintaining accuracy during the procedure. They may also provide surgeons with a novel monitoring method for complications such as haemorrhage, ischemia and oedema in the intraoperative setting. Specific modalities such as diffusion-weighted imaging (DWI) remain a highly sensitive method of MRI tissue characterisation for detecting ischaemic stroke [13]. An intraoperative MRI can utilise such a feature with cases demonstrating its function in detecting intra-operative ischaemic complications and subsequent management, e.g., recanalisation of the occlusion or administration of neuroprotective agents [14]. Other modalities, such as diffusion tensor imaging (DTI), can be utilised intraoperatively to assess the anatomy of white matter tracts, identifying normal structures as well as disruptions caused by tumour invasion or oedema [15]. Intraoperative MRIs utilising DTI have been used to detect white matter tract deformations and assist with their restoration to near normal position [15].

One metric for the usefulness of iMRI to the surgeon is the rate of further resection after intraoperative visualisation of residual tumours. This suggests that the surgeon was able to identify residual tumour margins on iMRI, which were not clear on gross inspection, allowing for a more complete resection that may not have otherwise been achievable.

Some studies in the literature [16,17,18,19,20] have investigated this topic; however, to our knowledge, no attempts have been made to compare the rates of further resection following iMRI across different tumour subtypes in adult patients, highlighting a significant research gap. The primary objective of our study was to evaluate the rates of further resection after iMRI detection of residual tumours across different brain tumour types. Our secondary objective was to analyse the trend in iMRI usage at our institution over a 17-year period.

## 2. Methods

To find neurosurgical cases within our institution which used iMRI, the intra-hospital database SurgiNet was searched, spanning September 2007 (induction of iMRI) to April 2023 (end capture date). Keywords such as “craniotomy”, “glioma”, “neurosurgery”, and “intraoperative magnetic resonance imaging” were used. Exclusion criteria incorporated paediatric patients (under 18 years), non-tumour resection cases and pregnant patients. The included cases were retrospectively analysed, and data extracted, including patient demographics (sex, age), tumour type, WHO grade, and the presence or absence of further resection following iMRI use. Further resection data were only available for the time periods 2007–2012 and 2018–2023.

**Figure 1:**
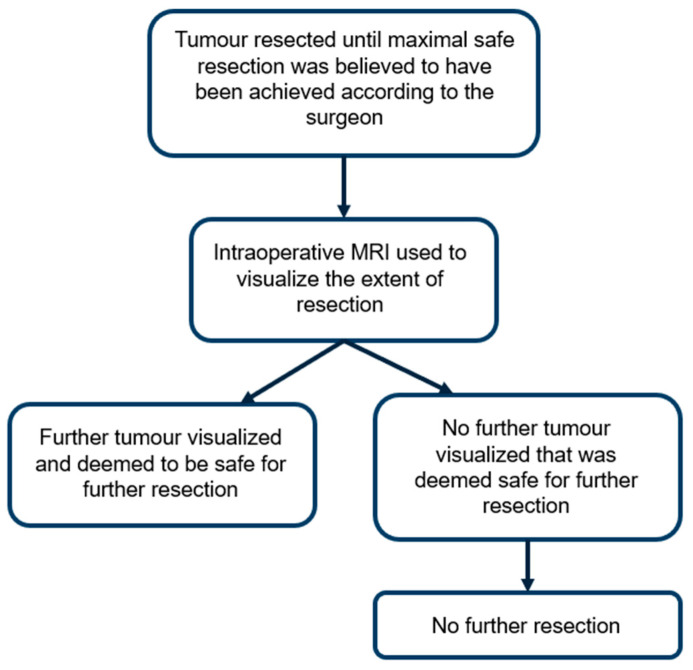
Integration of intraoperative MRI into the surgical workflow.

In our institution, intraoperative MRI was utilised when the surgeon determined that maximal safe resection had been achieved. Additional resection was performed only if residual tumour was detected and considered safe to remove. These further resections indicate tumour that was not identified through gross inspection but was deemed resectable. This workflow is outlined in Figure 1.

The primary outcome was the rate of further resection following iMRI use between tumour types. The secondary outcome was the change in rate of further resection between the two time periods (2007–2012 and 2018–2023). The secondary outcome was the duration of each iMRI case, measured from the patient’s entry into the iMRI theatre to their exit. Time data were available only for the period between 2017 and 2022.

Chi-square tests were used to compare both the rate of further resection between tumour types and the rate of further resection between time periods. Fischer’s test and Yates’ correction were not applied as each item in the Chi-square matrix had a value greater than five. A *p*-value less than 0.05 was considered statistically significant. Statistical analysis was performed using RStudio 2024.09.1.

## 3. Results

As summarised in Table 1, data from 486 patients were included in this study, with glioma resections forming the majority (270) of cases. A total of 273 participants (56%) were aged over 55 years old. A total of 252 participants (52%) were female. A total of 214 cases (44%) were classified as WHO Grade 1.

**Figure 2:**
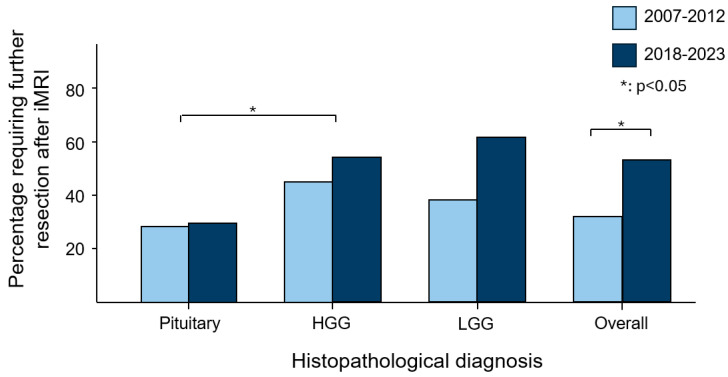
Rate of further resection by tumour subtype and time period.

The brain tumour type with the highest rate of further resection following iMRI were HGGs (47%), followed by LGGs (45%), and pituitary neuroendocrine tumours (PitNETs) (29%). There were no instances of further resection amongst meningiomas and metastases. The difference in proportions of further resections between HGGs and PitNETs was statistically significant (*p* = 0.04), but the other comparisons were not (LGG versus PitNET, *p* = 0.12; LGG versus HGG, *p* = 1.00). This is summarised in Table 2 and illustrated in Figure 2.

HGG, LGG, and PitNETs had higher rates of further resections in the second time period (2018–2023) compared to the first (2007–2012) (Table 2 and Figure 2). The difference in proportions within each tumour type across the time periods was not statistically significant. However, when tumour types were grouped together, the overall difference in proportions was statistically significant (*p* = 0.036).

**Figure 3:**
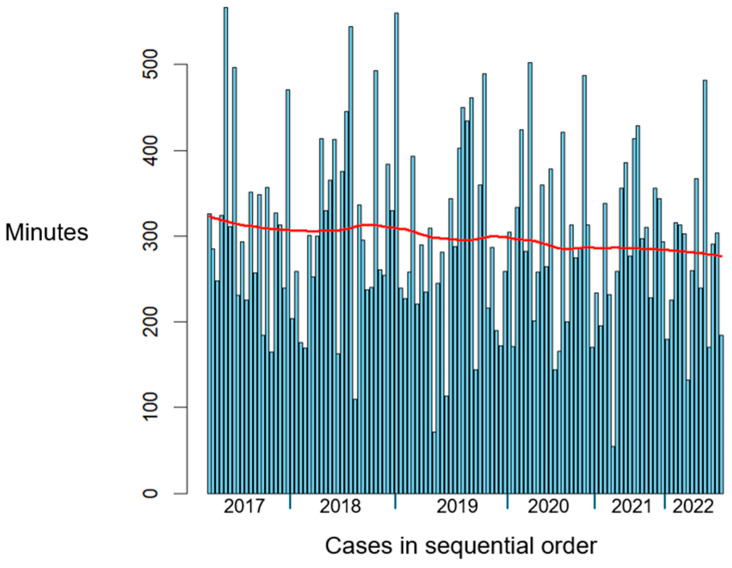
Minutes per iMRI case over time from 2017 to 2022. The red line represents a rolling average.

The time taken per iMRI case had an overall decreasing trend from 2017 to 2022, from a mean of 315 min in 2017 (SD = 98.8) to 281 min in 2022 (SD = 90.7). This is shown in Table 3 and illustrated in Figure 3.

## 4. Discussion

Neurosurgeons may utilise iMRI during surgery to detect residual tumours, enabling a more targeted approach to achieving maximal safe resection. In this study, we compared the frequency of further resection prompted by iMRI across various brain tumour subtypes. This offers valuable insight into the effectiveness of iMRI across different tumour subtypes from a surgeon’s perspective. As shown in Figure 1, intraoperative MRI appeared most useful in cases of HGG (47%) and LGG (45%), where there were higher rates of further resection compared to pituitary neuroendocrine tumours (29%; *p* = 0.04 reaching significance, and *p* = 0.12, respectively). This could be attributed to the challenge of differentiating glioma margins from normal brain tissue, particularly in cases of LGG, as well as the invasive nature of HGGs. There were no cases of further resection in meningiomas or metastases, likely due to these tumour subtypes typically having well-defined surgical planes, making iMRI less necessary in such situations. In addition, we observed a significantly higher reoperation rate in 2018–2023 compared to 2007–2012 (46% versus 33%; *p* = 0.036), which may reflect an initial learning curve followed by increased surgeon familiarity. There was a decreasing trend in the time taken per iMRI case from 2017 to 2022, suggesting that theatre and surgical staff became more efficient and familiar with iMRI use over time.

Our results are mostly in agreement with previous studies in the literature. The rates of further resection in glioma resections ranged from 26 to 47% [17,19,20]. Both our study and Hatiboglu et al. (2009) [17], with a reoperation rate of 47% (*n* = 46), place within the higher end of this range. Hatiboglu et al. speculate this to be a consequence of their selective use of iMRI for difficult cases involving infiltrative tumours in or around eloquent areas. This matched our experience and supports the use of iMRI for difficult glioma resections. The reoperation rate in our study for pituitary neuroendocrine tumours was 29%, higher than the rate of 20% found by Schwartz et al. (2006) [21]. This may be because Schwartz et al. exclusively used a two-nostril endoscopic endonasal approach, which may offer greater visualisation than the alternative microscopic approach. In our study, there was a mixture of the two methods, which may partially explain the higher rate of further resection.

Interestingly, Giordano et al. (2019) [16] found a 37% rate of further resection in their cohort study focusing on parasellar meningiomas (*n* = 19). This contrasts with our reoperation rate of 0% in six meningioma resections. This may be because our meningioma resections involved convexity meningiomas, which are easier to resect, while parasellar meningiomas are in a more anatomically challenging region due to their proximity to the cavernous sinus and difficult access. Indeed, where there was involvement of the cavernous sinus, Giordano et al. found a reoperation rate of 56%, with the difficulty in these procedures likely contributed by the proximity to cranial nerves and the cavernous segment of the internal carotid artery. Meanwhile, Kremer et al. (2006) [18] (*n* = 35) performed a study in paediatric brain tumours (with a varied case mix but predominantly LGGs) and found a reoperation rate of 60%, higher than any of the subtypes in our study with adult patients. This may be due to the proportionally greater impact of brain shift in paediatric resections where operations are performed in a smaller area.

While our study did not objectively quantify rates of gross total resection, there is evidence in the literature that iMRI increases the likelihood of achieving gross total resection. To assess the extent of resection intraoperatively, intra-operative fluorescent agents such as 5-aminolevulinic acid (5-ALA) or fluorescein sodium (FS) and intra-operative MRI have been the most evaluated tool in the literature, with the former being the current gold-standard [22,23]. A meta-analysis comparing 5-ALA, FS and iMRI for high-grade gliomas found that iMRI had the highest likelihood of achieving gross total resection (surface under cumulative ranking; S = 0.869, indicating a high likelihood of being the best available treatment) [24].

Despite the usefulness of iMRI for intraoperative surgical planning, the technology cannot be recommended for widespread use, whilst steep practical limitations remain. First, iMRI requires a specifically designed operating suite, MRI-compatible operating equipment, the MRI scanner itself, and ongoing maintenance and upgrade costs needed to meet technological developments [25]. The up-front installation of a single iMRI suite has been cited to cost USD 3 million [26]. Nonetheless, Abraham et al. performed a cost-effectiveness analysis and found that iMRI yielded an incremental benefit of 0.18 quality-adjusted life years (QALY) for an added cost of USD 13,447, which translates to a 99.95% chance of cost-effectiveness at a willingness-to-pay threshold of USD 100,000 per QALY [27]. Other practical considerations that must be navigated include imaging artefacts from the various retractors, Mayfield clamps, stabilising pins, surgical and anaesthetic probes, and MRI, ensuring compatibility of all operating personnel and equipment. Moreover, a retrospective study of patients undergoing awake craniotomy at a single Canadian institute reported both anaesthetic and surgical complications with the use of iMRI [28]. Five of twenty-three patients (22%) experienced significant nausea and vomiting and required conversion to general anaesthesia. Intra-operative complications such as seizures and speech deficits occurred in five of twenty-three patients (22%) operated on with iMRIs, compared to four of twenty-two patients (18%) who received awake craniotomy without iMRIs [28]. Whilst this result is not statistically significant and does not represent a valid comparison in a controlled trial, it raises the consideration that there may be other factors affecting the outcomes of operation involving iMRIs, e.g., the generally longer duration of surgery and anaesthesia with iMRIs.

The present study had some limitations. Our primary outcome to determine the usefulness of iMRI was the rate of further resection after iMRI. However, this is a subjective metric affected by surgeon preference. Surgeons may be biased to image early and check the progress of resection due to knowing that iMRI is available. Therefore, the presence of further resection when iMRI is available would not necessarily indicate that a more complete resection had occurred compared to if iMRI were not available. A more objective outcome would have been the rate of complete resection, but unfortunately, these data were not available to us. Moreover, our data regarding the rate of further resection relies on the accuracy of the documented operation report. A further limitation is the absence of data on postoperative complications, which would be needed to confirm that iMRI-guided further resection was safe. While most cohort studies using iMRI found no surgical complications related to its use [16,17,18,20,21] there have been some reports of non-surgical hazards such as thermal injuries to the patient from induced currents in ECG leads. Overall, iMRI is reasonably safe in the context of precautionary checklists such as the Zurich Checklist for Safety.

## 5. Conclusions

Intraoperative MRI appears to be particularly valuable for surgeons in enhancing the extent of safe tumour resection in gliomas compared to other brain tumour types. Intraoperative MRI may also be useful in procedures targeting locations that are difficult to access or have proximity to critical structures, such as resections of PitNETs involving the cavernous sinus. Surgeons increasingly alter their surgical strategy using iMRI over time, which may reflect increasing familiarity and comfort with the technology.

## Figures and Tables

**Table 1:** Demographic characteristics of intraoperative MRI dataset.

Variable	All Types	Glioma	PitNET	Metastases	Meningioma
	486	270	194	14	8
Age					
55 or less (*n* [%])	213 (44%)	157 (58%)	52 (27%)	1 (7%)	3 (38%)
Over 55 (*n* [%])	273 (56%)	113 (42%)	142 (73%)	13 (93%)	5 (62%)
Gender					
Female (*n* [%])	252 (52%)	130 (48%)	107 (55%)	7 (50%)	8 (100%)
Male (*n* [%])	234 (48%)	140 (52%)	87 (45%)	7 (50%)	0 (0%)
Awake craniotomy	5	5	-	-	-
WHO grade					
1	214	16	194	-	4
2	79	79	-	-	-
3	93	93	-	-	-
4	77	77	-	-	-
NOS	15	5	-	10	-

PiTNET, pituitary neuroendocrine tumour; NOS, not otherwise specified.

**Table 2:** Proportion of cases that underwent further resection after iMRI by case type and time period.

Case Type	2007–2012	2018–2023	*p*-Value	Overall
PitNET	25/86 (29%)	10/33 (30%)	0.956	35/119 (29%)
HGG	27/61 (44%)	14/26 (54%)	0.412	41/87 (47%)
LGG	19/50 (38%)	14/23 (61%)	0.07	33/73 (45%)
Meningioma	0/6	0/0	-	0/6
Metastasis	0/11	0/0	-	0/11
Overall	71/214 (33%)	38/82 (46%)	0.036	109/296 (37%)

PiTNET, pituitary neuroendocrine tumour; HGG, high-grade glioma; LGG, low-grade glioma.

**Table 3:** Minutes taken per iMRI case from 2017 to 2022.

Year	Minutes Per Case (SD) *
2017	315 (98.8)
2018	306 (105)
2019	294 (115)
2020	294 (102)
2021	291 (91.7)
2022	281 (90.7)

*: Time measured from the time of entry into the theatre to the time of exit from the theatre.

## Data Availability

The original contributions presented in this study are included in the article. Further inquiries can be directed to the corresponding author(s).
